# Association between NOS3 *G894T, T-786C* and 4a/4b Variants and Coronary Artery Diseases in Iranian Population

**Published:** 2018-12

**Authors:** Hamid Reza JOSHAGHANI, Aref SALEHI, Esmaeal SAMADIAN, Roghayeh GHARAEI, Ali Reza AHMADI

**Affiliations:** 1. Biochemistry & Metabolic Disorders Research Center, Golestan University of Medical Sciences, Gorgan, Iran; 2. Golestan Research Center for Ischemic Disorders, Golestan University of Medical Sciences, Gorgan, Iran; 3. Dept. of Biotechnology, Faculty of Advanced Medical Technologies, Golestan University of Medical Sciences, Gorgan, Iran; 4. Laboratory Science Research Center, Golestan University of Medical Sciences, Gorgan, Iran

**Keywords:** Endothelial nitric oxide synthase, Coronary artery disease, Polymorphism

## Abstract

**Background:**

Endothelial nitric oxide synthase, encoded by *NOS3*, produces an atheroprotective metabolite. The *G894T, T-786C* and 4a/4b variants of this gene associated with increased risk for coronary artery diseases (CAD) have been evaluated in different populations worldwide, and inconsistent results have been obtained. We investigated the association between these three polymorphisms and presence of CAD in Iranian individuals.

**Methods:**

Overall, 234 people including angiography-positive patients from Amir-Almomenin Hospital (Heart Center), Kordkoy City, Golestan Province, northern Iran in 2016, angiography-negative subjects and healthy individuals from north of Iran were genotyped for the *G894T* and *T-786C* variations by PCR-RFLP, and 4a/4b VNTR only by PCR.

**Results:**

The genotype distribution and allelic frequencies for the three variants tested were not dramatically different between CAD and control subjects and also between CAD patients and people with pains and symptoms very similar to CAD but no stenosis (*P*>0.05). Moreover, the odds ratio for CAD related to the G894T (OR=1.09, 95% CI=(0.60–2.00), T-786C (OR=1.04, 95% CI=(0.57–1.89) and 4a/4b (OR=1.75, 95% CI=(0.92–3.32) variants did not show statistical significance. Similarly, the odds ratio for stenosis confirmed by angiography related to the 894T (OR=1.03, 95% CI= (0.61–1.74), −786C (OR=0.90, 95% CI=(0.54–1.50) and 4b (OR=1.64, 95% CI=(0.92 –2.93) alleles were not significant.

**Conclusion:**

*G894T*, *T-786C* and 4a/4b variants were not associated with risk for CAD and occurrence of angiography-assessed stenosis in Northern Iranian population (*P*>0.05). These alleles might be population-specific and not to be associated with their corresponding gene pool. However, further analysis is required to clarify other CAD-correlated markers in our community.

## Introduction

Coronary artery disease (CAD) or atherosclerosis is the most common cause of death worldwide, with vast inter-ethnic differences. Interactions between both environmental and genetic contributors confer ethnic geographical variations in disease fashions (1, 2). Nowadays, production of nitric oxide (NO), a vasodilator catalyzed by the endothelial NO synthase (eNOS), is a critical process in the endothelial activity preventing the atherosclerotic events. NO has atheroprotective impacts as it declines vascular balance, blood pressure, lipoprotein particle oxidation, platelet aggregation and, also, inhibits leukocyte adhesion and movement through the endothelial tissue. Due to the decreased expressions of the eNOS mRNA and protein in human atherosclerotic vessels, eNOS is regarded as a putative candidate for the susceptibility to CAD (2, 3). Hence, any inherited changes in its corresponding gene that possibly lead to abnormalities in the transcriptional and post-transcriptional regulations may implicate in the development of cardiovascular dysfunction (3–5).

The eNOS protein has a molecular weight of 135 kDa, consisting 1203 amino acids in length, and it is encoded by *eNOS* or *NOS3* gene, which is 21 kb with 26 exons extending through the chromo-some 7q35–36 (6). However, several population genetic types of research in recent years suggested that different polymorphisms in NOS3 can be the promising CAD-correlated markers, but different results have been obtained. Among these controversial polymorphisms, systematic reviews and meta-analyses have indicated that two single-nucleotide polymorphisms (SNPs) related to eNOS protein expression, including rs1799983 (*894G>T*, *Glu298Asp*) in exon 7 and rs2070744 (−*786T>C*) in the promoter region and the variable number of tandem repeats (VNTR) in intron 4 (rs61722009 4a/4b) have been extensively screened in the *eNOS* gene (7–10).

Although these three clinically relevant variants are also known as CAD susceptibility, these are not consistent in all populations. Since few evidence of the relationships between these polymorphisms (rs1799983, rs2070744, and rs61722009 alleles) and CAD have been reported in populations from the west of Asia, we have decided to study these alleles and their relations with CAD in Iranian populations.

## Materials and Methods

### Study population

The subjects enrolled in this case-control study, comprised (i) 93 cases with stenosis of at least 50% in a major coronary artery who visit angiography section in Amir-Almomenin Hospital (Heart Center) in Kordkoy City of Golestan Province, northern Iran in 2016; (ii) 48 individuals with pains and symptoms very similar to CAD but no stenosis, as confirmed by angiography and (iii) 93 healthy controls from unrelated families living in the Golestan Province (North of Iran). All subjects gave written informed consent to participate. In general, blood samples were taken from three groups in our study.

### Genotyping analysis

Genomic DNA samples of all individuals were isolated from whole blood collected with anticoagulant (EDTA, 15% w/v), using a ‘salting out’ method (11). PCR were carried out to amplify the eNOS rs1799983 (G/T), rs2070744 (T/C) and rs61722009 (4a/4b) regions. Then, a restriction enzyme-based method was applied to genotype the rs1799983 and rs2070744 variants and the digestion patterns were analyzed by Agarose-gel electrophoresis (12, 13). Finally, the number of 27 bp-tandem repeats of 4a/4b allele were also detected on Agarose-gel electrophoresis (14) (Table 1).

**Table 1:** Primers and enzymes used for screening NOS_3_ polymorphisms

***Gene***	***Amplicon***	***Primer sequences***	***Annealing temperature (°C)***	***Product size (bp)***	***Enzyme***	***Fragments size (bp)***
*NOS_3_*	G894T	F: CAT GAG GCT CAG CCC CAG AAC R: AGT CAA TCC CTT TGG TGC TCA C	61	206	MboI (New England Biolabs)	119 + 87
*NOS_3_*	T-786C	F: ATG CTC CCA CCA GGG CAT CA R: GTC CTT GAG TCT GAC ATT AGG G	51	236	NgoMIV (New England Biolabs)	203 +33
*NOS_3_*	4a4b	F: AGG CCC TAT GGT AGT GCC TT R: TCT CTT AGT GCT GTG GTC AC	49	420 - 393	------	------

### Statistical analysis

The variants under investigation were evaluated for deviation from Hardy–Weinberg equilibrium by comparing observed and expected genotype frequencies by means of Chi-square test in the control groups. The statistical difference in genotype distribution and allele frequencies in both control and case subjects was assessed by using standard 2 × 2 χ^2^ test. Odds ratios (ORs) and confidence intervals (CIs) were calculated and a *P*-value of 0.05 was determined as significant.

## Results

The CAD study group (n= 93), angiography-negative subjects (n= 48) and unrelated healthy controls (n = 93) were of similar sex and age with 58 ± 5 yr (Mean± SD). Allele and genotype frequencies of the analyzed samples of the *eNOS* rs1799983 G/T, rs2070744 T/C, and rs61722009 4a/4b polymorphisms are depicted in Tables 2–4. Deviation from Hardy–Weinberg equilibrium was not found with regard to the distribution of our studied genotypes and allele frequencies in all groups (*P*>0.05). Accepting a recessive model of inheritance, we found that the mutant genotypes were not common in CAD patients (894T/T: 1.07%, −786C/C: 0% and 4b/4b: 1.07%) as compared with control individuals (894T/T: 2.15%, −786C/C: 4.3% and 4b/4b: 1.07%) and the differences were not statistically significant. The observed differences were calculated with a 2 × 2 χ^2^ test. So, it cannot be considered that there are relationships between the mutant genotypes and Northern Iranian patients with CAD (OR=0.51, 95% CI= (0.01–6.99), OR=0, 95% CI= (0–1.18), OR=1.18, 95% CI= (0.02–46.89) respectively).

**Table 2:** Genotype and allele distributions of polymorphisms of endothelial nitric oxide synthase rs1799983 gene in CAD patients (n=93), angiography-negative subjects (n=48) and controls (n=93) in North Iranian^*^

***NOS3 G894T (rs1799983)***								
***Characteristic***	***Case n (%)***	***Control n (%)***	***Odds ratio (95% CI)***	***P value***	***Case n (%)***	***Angiography negative subjects n (%)***	***Odds ratio (95% CI)***	***P value***
Genotypes								
GG	58(62.36)	60 (64.51)	1	-	58 (62.36)	29 (60.41)	1	-
TG	34(36.55)	31(33.33)	1.13 (0.61 – 2.08)	0.686	34 (36.55)	18 (37.5)	0.94 (0.45 – 1.97)	0.875
TT	1(1.07)	2 (2.15)	0.51 (0.01 – 6.99)	0.650	1 (1.07)	1 (2.08)	0.50 (0.01 – 20.14)	0.674
TG + TT	35(37.63)	33(35.48)	1.09 (0.60 – 2.00)	0.763	35 (37.63)	19 (39.5)	0.92 (0.44 – 1.90)	0.821
Alleles								
G	150(80.64)	151(81.18)	1	-	150 (80.64)	76 (79.16)	1	-
T	36(19.35)	35 (18.81)	1.03 (0.61 – 1.74)	0.896	36(19.35)	20 (20.83)	0.91 (0.49 – 1.70)	0.764

* Two-tailed chi square (χ^2^) test significant at <0.05; CI: confidence interval

**Table 3:** Genotype and allele distributions of polymorphisms of endothelial nitric oxide synthase rs2070744 gene^*^ in CAD patients (n=93), angiography-negative subjects (n=48) and controls (n=93) in North Iranian^*^

***NOS3 T-786C (rs2070744)***								
***Characteristic***	***Case n (%)***	***Control n (%)***	***Odds ratio (95% CI)***	***P value***	***Case n (%)***	***Angiography negative subjects n (%)***	***Odds ratio (95% CI)***	***P value***
Genotypes								
TT	56 (60.2)	57(61.3)	1	-	56 (60.2)	30 (62.5)	1	-
TC	37 (39.8)	32(34.4)	1.17 (0.64 –2.15)	0.599	37 (39.8)	16 (33.33)	1.23 (0.59 – 2.62)	0.576
CC	0 (0)	4(4.3)	0 (0 – 1.18)	0.070	0 (0)	2 (4.16)	0 (0 – 1.95)	0.129
TC + CC	37 (39.8)	36(38.7)	1.04 (0.57 – 1.89)	0.882	37 (39.8)	18 (37.5)	1.10 (0.53 – 2.28)	0.798
Alleles								
T	149 (80.1)	146(78.5)	1	-	149 (80.1)	76 (79.16)	1	-
C	37 (19.9)	40(21.5)	0.90 (0.54 – 1.50)	0.703	37 (19.9)	20 (20.83)	0.94 (0.51 – 1.76)	0.846

* Two-tailed chi square (χ^2^) test significant at <0.05; CI: confidence interval

**Table 4:** Genotype and allele distributions of polymorphisms of endothelial nitric oxide synthase Intron 4 polymorphism in CAD patients (n=93), angiography-negative subjects (n=48) and controls (n=93) in North Iranian^*^

***Intron 4 polymorphism of NOS3***								
***Characteristic***	***Case n (%)***	***Control n (%)***	***Odds ratio (95% CI)***	***P value***	***Case n (%)***	***angiography-negative subjects n (%)***	***Odds ratio (95% CI)***	***P value***
Genotypes								
bb	59 (63.44)	70(75.26)	1	-	59 (63.44)	35 (72.91)	1	-
ab	33 (35.48)	22(23.65)	1.77 (0.93 – 3.40)	0.079	33 (35.48)	12 (25)	1.63 (0.74 – 3.65)	0.224
aa	1 (1.07)	1(1.07)	1.18 (0.02 – 46.89)	0.916	1 (1.07)	1 (2.08)	0.59 (0.01 – 23.77)	0.750
bb + ab	34(36.56)	23(24.7)	1.75 (0.92 – 3.32)	0.083	34(36.55)	13 (27.08)	1.54 (0.72 – 3.40)	0.265
Alleles								
b	151 (81.18)	163(87.6)	1	-	151 (81.18)	82 (85.41)	1	-
a	35 (18.81)	23(12.36)	1.64 (0.92 – 2.93)	0.088	35 (18.81)	14 (14.58)	1.35 (0.69 – 2.73)	0.382

* Two-tailed chi square (χ^2^) test significant at <0.05; CI: confidence interval

Patients with CAD also did not commonly show the mutant alleles (894 T: 19.35%, −786 C: 19.9% and 4b: 18.81%) compared to controls (894 T: 18.81%, −786C: 21.5% and 4b: 12.36%). Therefore, there is no association between the mutant alleles and CAD in patients of Northern Iranian origin.

In the same form, the mutant genotypes were not prevalent in CAD patients as compared with the subjects having no stenosis in coronary artery (894 T/T: 2.08%, −786C/C: 4.16% and 4b/4b: 2.08%) and the genotype distributions and allele frequencies for the three variants were not dramatically different between these two groups. The obtained differences cannot be assumed that there are relationships between mutant genotypes and the stenosis in our people.

**Fig. 1:**
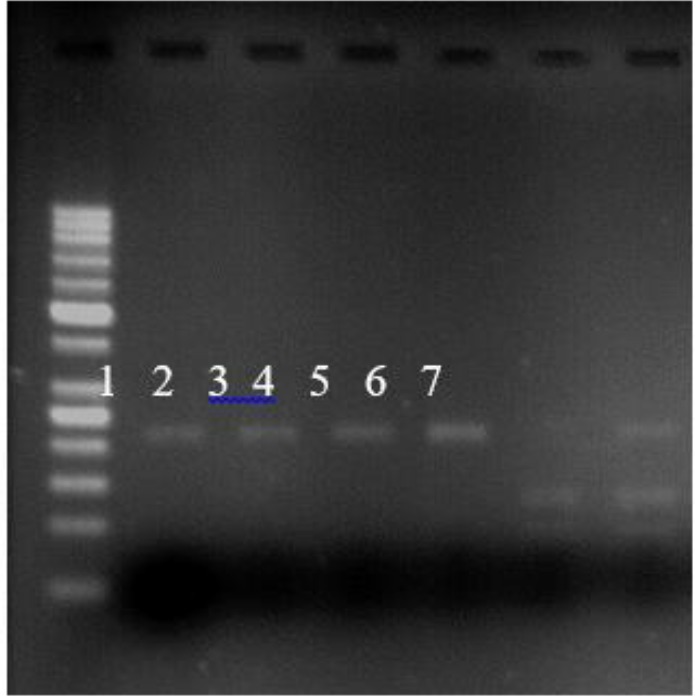
Analysis of the G894T polymorphism of the eNOS gene. Lane 1: 50 base pair (bp) DNA Ladder, Lane 2,3,4,5 GG genotype, Lane 6 TT genotype, Lane 7 GT genotype

**Fig. 2:**
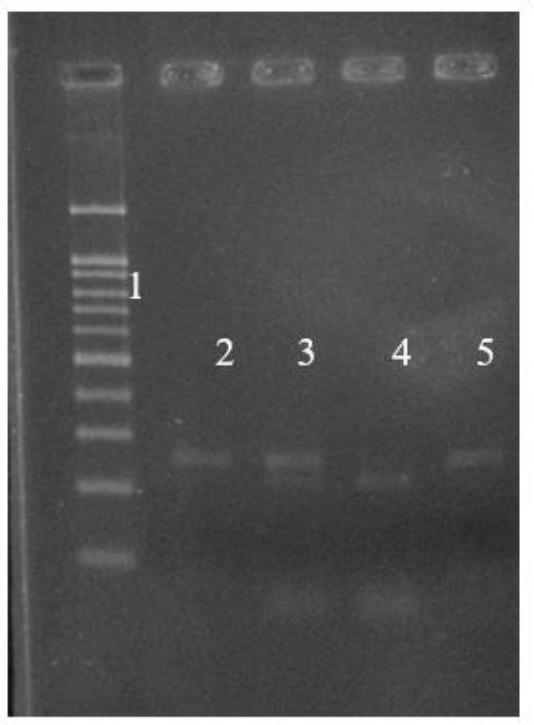
Analysis of the T786 polymorphism of the eNOS gene. Lane 1: 100 base pair (bp) DNA Ladder, Lane 2, 5 TT genotype, Lane 3 :CT genotype, Lane 4 CC genotype

**Fig. 3:**
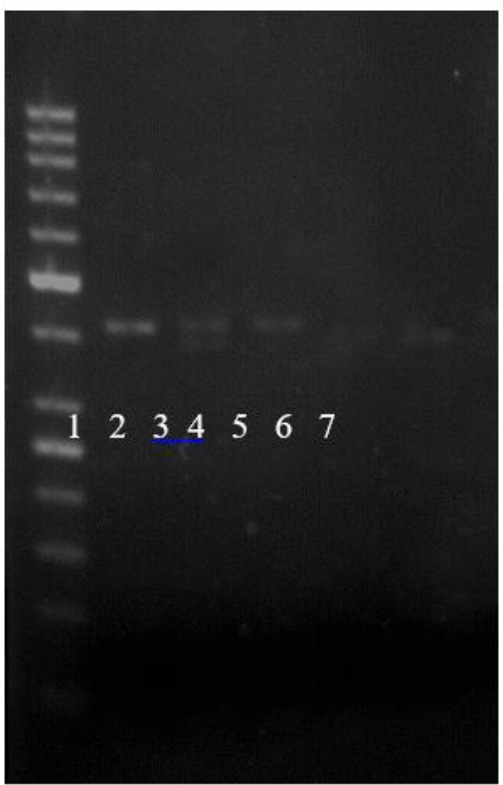
Analysis of the variable number of tandem repeats (VNTR) polymorphism in intron 4 of the eNOS gene. The 420-bp band indicates five 27-bp repeats, and the 393-bp band indicates four 27-bp repeats. Lane 1: 50 bp DNA Ladder Lane 2, 3 4b/4b, Lane 5, 6: 4a/4a Lane 3: 4b/4a

The mutant alleles were not frequently found in patients with CAD compared to the subjects with no confirmed stenosis (894 T: 20.83%, −786C: 20.83% and 4b: 14.58%) (Figs. 1–3). Thus, there is no association between the mutant alleles and the stenosis in patients of our origin.

## Discussion

The relationships between different *eNOS* diversities, as candidate genetic markers, and cardiovascular disease in different ethnic groups have been recently evaluated in various investigations. Meta-analyses of the conflicting results gained from these clinical genomic studies also revealed that this gene could have ethnic-dependent variations with a higher prognostic value for CAD risks such as rs1799983, rs2070744 and rs61722009 polymorphisms (7–9). In this issue, rs1799983 (*G894T*) and rs61722009 (4b/a) variants are supposed to carry the highest risk for CAD in the Middle Easterners though, rs2070744 (*T-786C*) and its minor allele have the greatest status of correlation among individuals of Asian ancestry (9). Moreover, an updated pooled analysis suggested that 4b/a variability might be a risk factor for developing CAD, especially in African communities (7).

Regarding Iranians, several association studies demonstrated that the *eNOS* rs1799983 polymorphism could affect susceptibility to CAD in populations from center of Iran (15–17). Consistently, the risk of atherosclerosis has been increased by the presence of T allele of the G894T variant and the levels of low-density lipoprotein cholesterol and triglycerides tended to be higher in patients carrier for 894T allele compared to those with 894G allele from west of this country (18). Although, a variety of reports from UK (19), Hungry (20), Taiwan (21), Egypt (22) and Tunis (23) confirmed this data. However, similar observations were not found in Australian (24, 25), Chilean (26), Turkish (27, 28) and Polish (28) populations, these inconsistencies were also verified in Asians (29), Indian (30) and Korean (5) ethnicities.

In contrast to the finding from patients of central parts of Iran (16), association between −786C allele and lower grade of the vessel dilation factor have been shown in patients suffering CAD from north-west of Iran (31). Comparatively, incompatible with information from, Japanese (32), Korean (33) and north-Indian (34) communications, lack of CAD-susceptibility relevance for *T-786C* has been represented among Chilean (26), and Saudi people (29). In relation to rs61722009, 4a allele of this mutation has been recognized to be a major risk indicator for coronary heart disease according to numerous testing in patients from center of Iran (35–37). Besides, these considerations were same as those from southern Turkey (38), Chilia (26), and Korea (33), but not supported by other works (39–41).

Interestingly, NO levels were indicated to be increased in CAD patients than in controls from south India. The homozygous mutant (TT) genotype of *G894T*, but not 4b/a variant, were associated with genetic susceptibility to coronary artery disease. On the other hand, both the *eNOS* 4b/a and G894T polymorphisms were not linked to plasma NO levels in their studied subjects (39).

In the current survey, there were no relations between rs1799983, rs2070744 and rs61722009 and CAD in Northern Iranian population. Therefore, these three variants might not be genius alleles related to CAD in Iranians and also, there are distinct population-specific differences in the prevalence of these alleles. Furthermore, these polymorphic alleles might not be significantly related to angiography-assessed stenosis as a result of an unclear mechanism. The Iranian population is composed of different ethnic groups, so, identifying alleles that have lower frequency in this population is hardly possible. The gene pool in our studied population may specifically have other CAD-susceptibility alleles because of migration, recombination and so on. Thus, further attempts are needed to prove our study.

These challenging findings may reflect epigenetic effects of these alleles in different populations. To our knowledge, polymorphisms −786T/C and 4b/a respectively located in the promoter and intronic region of *eNOS* could contribute to the risk of cardiovascular disease, lower *eNOS* mRNA, and serum nitrite/nitrate levels have been observed in subjects with the −786C or 4a allele (40, 42). Notably, these two variants jointly influenced the transcriptional efficiency of *eNOS* in a haplotype-dependent manner (41) and that the 4b/a polymorphism affected the bioavailability and activity of NO via linkage disequilibrium with T-786C and G894T variants (40). 4a allele is perhaps in linkage disequilibrium with other important alterations in regulatory regions of the NOS3 gene (10). Moreover, the T allele of rs1799983 (G894T) polymorphism in exon 7, as a structural substitution of the eNOS protein (E298D), causes the *eNOS* more sensitive to intra-cellular proteolytic cascades. This may generate N-terminal 35 kDa and C-terminal 100 kDa fragments from the mature protein and a reduction in its functionality and NO production, which subsequently elevates the CAD risk (43–45).

## Conclusion

Although many CAD-associated genetic risk factors have been characterized by now many efforts remained to be attempted to make a gene map for these markers. At the end, we cannot turn a blind eye to environmental factors in CAD development and progression.

## Ethical considerations

Ethical issues (Including plagiarism, informed consent, misconduct, data fabrication and/or falsification, double publication and/or submission, redundancy, etc.) have been completely observed by the authors.
